# Morpho-anatomical studies of family lamiaceae species of district Lahore, Punjab: a revision to flora of Pakistan

**DOI:** 10.1186/s12870-024-05358-0

**Published:** 2024-07-22

**Authors:** Javaria Majeed, Shabnum Shaheen, Muhammad Waheed, Moneeza Abbas, Nadia Ghani, Muhammad Ashfaq, Abeer Hashem, Ajay Kumar, Elsayed Fathi Abd-Allah

**Affiliations:** 1 Department of Botany, Lahore College for Women University, Lahore, 54000 Pakistan; 2 Department of Botany, Faculty of Life Sciences, University of Okara, Okara, 56300 Pakistan; 3 Department of Ethnobotany, Institute of Botany, Ilia State University, Tbilisi, 0162 Georgia; 4 Department of Environmental Sciences, Lahore College for Women University, Lahore, 54000 Pakistan; 5 Department of Plant Breeding and Genetics, Faculty of Agricultural Sciences, University of the Punjab, Lahore, 54590 Pakistan; 6 Botany and Microbiology Department, College of Science, King Saud University, P.O. Box. 2460, Riyadh, 11451 Saudi Arabia; 7 Amity Institute of Biotechnology, Amity University, Sector-125, Noida, Uttar Pradesh 201313 India; 8 Plant Production Department, College of Food and Agricultural Sciences, King Saud University, P.O. Box. 2460, Riyadh, 11451 Saudi Arabia

**Keywords:** Lamiaceae, Light microscopy, Scanning electron microscopy, Paracytic stomata, Peltate and glandular trichomes

## Abstract

**Background:**

This study was aimed to determine the taxonomic position and delimitation of fifteen Lamiaceae taxa using leaf epidermal morpho-anatomical features in Lahore. A main objective of the study was also the revision and upgradation of Lamiaceae taxa in the flora of Pakistan, as no details of studied species are found in the flora of Pakistan.

**Methods:**

The examination of significant anatomical parameters, such as epidermal cell shape and size, stomatal types, guard and subsidiary cells shape and size, stomatal cavity size, trichome size and shape, oil droplets, crystals, and secretory cavity characteristics were studied using light microscopic (LM) and scanning electron microscopic (SEM) techniques. Among all the studied Lamiaceae species, these anatomical features varied significantly. Principal component analysis and correlation were done to distinguish the species’ similarities.

**Results:**

Most species had pentagonal and hexagonal epidermal cells with straight anticlinal wall thickness. On the adaxial surface, paracytic stomata were found in *Ocimum basilicum* L. and *Rosmarinus officinalis* L. Diacytic stomata was observed in *Ajuga reptans* L. and anisocytic stomata in *Galeopsis tetrahit* L. In the abaxial surface, trichomes were present in five species, i.e., *Mentha suaveolens* Ehrh. *A. reptans, Thymus vulgaris L., M. haplocalyx*, and *Salvia splendens* Ewat. In *S. splendens*, peltate and glandular trichomes were seen whereas, in other species, trichomes were long, unbranched glandular and had tapering ends. In adaxial side trichomes were present only in *M. suaveolens, A. reptans, S. bazyntina, O. basciculum, S. splendens, S. officinalis, S. rosemarinus*. In other species, trichomes were absent on the adaxial surface. In abaxial view, *M. suaveolens* had the largest length of trichomes, and *O. basciculum* had the smallest. *S. splendens* L. had the largest trichome width, while *T. vulgaris* had the smallest.

**Conclusion:**

Hence, according to these findings, morpho-anatomical traits are useful for identifying Lamiaceae taxa. Also, there is a need of upgradation and addition of studied taxa in flora of Pakistan comprehensively.

## Introduction

The Lamiaceae or Labiatae, commonly known as the mint family, comprises flowering plants. Previously, it was thought to have close associations with Verbenaceae [1]. However, phylogenetic investigations carried out in the 1990s revealed that several genera traditionally categorized under Verbenaceae actually belong to the Lamiaceae family. The present understanding suggests that the current taxonomy of Verbenaceae may not necessarily indicate a closer relationship to Lamiaceae than other families within the Lamiales order [2]. Furthermore, the closest family to Lamiaceae within the Lamiales order remains undetermined. This family is distributed worldwide [3, 4].

The Lamiaceae family is renowned for its abundant aromatic plants, which encompass numerous well-known culinary herbs such as basil, mint, rosemary, sage, savory, marjoram, oregano, thyme, lavender, and perilla. Some are also cultivated for ornamental foliage, like coleus. The leaves of these plants emerge in an opposite pattern, with each pair positioned at right angles to the preceding one (known as decussate) or in whorls [5, 6]. The plant family Lamiaceae is renowned for its valuable medicinal and aromatic herbs such as lavender, mint, oregano, sage and thyme. It serves as a rich reservoir of essential oils utilized in the food, pharmaceutical and cosmetic industries. Despite their botanical diversity and traditional significance, species within the Lamiaceae family exhibit mechanisms that contribute to the improvement of numerous diseases, including cardiovascular ailments [7–9].

The scanning electron microscopy (SEM) development has facilitated the addition of a broader range of characters for morphological, anatomical and palynological investigations. The recent application of SEM has expanded the scope of systematic and evolutionary inquiries across numerous taxa. A significant outcome of these studies has been the revision of relationships proposed by fundamental and traditional data aligning with the modern taxonomic data. In addition to its considerable economic and medicinal value, the Labiatae family makes a substantial contribution to endemic flora.

By elucidating the morpho-anatomical characteristics of plant species, researchers can gain insights into their ecological adaptations, distribution patterns, and responses to environmental changes. This information is crucial for effective conservation planning and management strategies. In Pakistan, where biodiversity is rich but often threatened by habitat loss, climate change, and other anthropogenic activities, understanding the intricacies of plant morphology and anatomy can aid in identifying key species for conservation priority. Furthermore, on a global scale, morpho-anatomical studies contribute to the broader understanding of plant diversity and evolution. They help in clarifying species boundaries, resolving taxonomic uncertainties, and identifying evolutionary relationships among different taxa. Incorporating a discussion on the broader conservation implications of morpho-anatomical studies in the research findings not only highlights their scientific significance but also underscores their practical relevance in addressing pressing environmental challenges both locally in Pakistan and on a global scale.

PCA is chosen for its ability to reduce the dimensionality of the dataset while preserving most of the variability. In our study, where we have multiple anatomical parameters measured across different samples, PCA allows us to identify the most significant patterns or clusters within the data.Pearson correlation, on the other hand, is selected to explore the relationships between different anatomical parameters quantitatively. It helps us understand how changes in one parameter correlate with changes in another, providing valuable insights into potential associations or dependencies.Pearson correlation allows us to quantify the strength and direction of relationships between anatomical parameters, aiding in the identification of potential biomarkers or key features associated with certain traits or conditions. While PCA is effective in dimensionality reduction, it may obscure subtle variations or outliers in the data. Careful interpretation of the results is necessary to avoid oversimplification or misrepresentation of the underlying patterns. Pearson correlation assumes linear relationships between variables and may not capture non-linear associations effectively. Additionally, it is sensitive to outliers and may not be suitable for skewed or non-normally distributed data.

The key objectives of this investigation were to document and visually represent the morpho-anatomical traits of leaves in Lamiaceae species, using both light and scanning electron microscopic techniques.

## Results

Current studies indicate variations among different species of family Lamiaceae having high taxonomic values. Following are the taxa selected for research work. *Mentha suaveolens* ehrh, *Mentha haplocalyx, Mentha piperata*, *Ajuga reptans, Coleus scutellarioides, Origanum vulgare, Oscimum basiicum, Salvia rosemarinus, Salvia splendens, Salvia officinalis, Stachys byzantine, Thymus vulgare* and *Tactona grandis* (Tables 1 and 2).

**Table 1:** Quantitative anatomical variations among studied Lamiaceae Taxa (Abaxial surface)

Plant Name	Epidermal cell length µm	Epidermal cell width µm	Stomatal cells length µm	Stomatal cells width µm	Guard cells length µm	Guard cell width µm	Subsidiary cells length µm	Subsidiary cells width µm	Trichome length Μm	Trichomewidth µm
*Mentha suaveolens* Ehrh. LCW-501	170.9 μm (148.6 − 203.7 μm)	210.9 μm (70.5 – 168.8 μm)	133.0 μm (123.4– 132.5 μm)	134.9 μm (122.6 μm 153.1 μm)	123.6 μm (112.7–138.2 μm)	64.5 μm (53.7 μm – 78.4 μm	175.3 μm (115.5–261.0 μm)	78.7 μm (51.0 – 118.6 μm)	67.9 μm (65.2 μm 69.0 μm)	92.3 μm (82.3–107.2 μm).
*Ajuga reptans* L. LCW- 502	245.6 μm (202.6–290.3 μm)	150.1 μm (132.3 – 174.8 μm)	160.6 μm (156.5– 167.3 μm)		155.0 μm (134.0– 170.5 μm)	143.1 μm (139.2µm161.5 μm)	295.7 μm (244.3–328.9 μm)	50.9 μm (45.0–53.9 μm)	145.0 μm (104.4–182.3 μm)	93.3 μm
*Coleus scutellaroides* L. LCW-503	240.1 μm (206.2–291.8 μm)	98.7 μm (78.6–114.2 μm)	101.4 μm (68.2- 133.6 μm)	53.4 μm (34.5–68.9 μm)	127.3 μm (102.6–142.8 μm)	50.2 μm (45.6–56.2 μm)	114.4 μm (102.3–124.3 μm)	103.3 μm (96.3 μm – 112.5 μm	0	0
*Stachys bazantina* K.Koch LCW-504	192.1 μm (178.0- 205.7 μm)	136.7 μm (118.9 –145.7 μm)	204.2 μm (196.3–214.5 μm)	102.9 μm (94.1 –111.1 μm)	134.5 μm (96.9 μm 174.3 μm)	29.1 μm (24.6- 32.9 μm)	196.7 μm (152.4–231.5 μm)	155.2 μm (99.8–207.3 μm)	0	0
*Origanum vulgare* L. LCW-505	104.9 μm (90.4–113.5 μm)	71.9 μm (63.0–78.5 μm)	98.7 μm (84.0–115.5 μm)	87.2 μm (80.2 – 91.4 μm)	111.3 μm (98.5- 132.6 μm)	29.2 μm (19.6 – 41.8 μm)	157.3 μm (102.8 – 245.3 μm)	148.7 μm (96.1 **–** 215.2 μm).	0	0
*Thymus vulgaris* L. LCW-506	157.9 μm (136.6 – 173.6 μm)	82.7 μm (55.4 – 101.1 μm)	127.0 μm (112.6– 143.2 μm)	91.8 μm (65.3–113.4 μm)	111.3 μm (98.5- 132.6 μm)	29.2 μm (19.6 – 41.8 μm)	157.3 μm (102.8–245.3 μm)	148.7 μm (96.1 **–** 215.2 μm).	48.2 μm (40.2–57.3 μm)	50.9 μm (45.0–53.9 μm)
*Oscimum basiculum* L.	153.1 μm (131.0–174.6 μm)	106.2 μm (78.9 – 146.7 μm)	104.6 μm (97.5 – 111.6 μm)	92.9 μm (67.9 –112.4 μm)	114.4 μm (89.6- 156.4 μm)	30.4 μm (26.4 – 35.2 μm)	203.6 μm (124.9–264.3 μm)	216.8 μm (196.4 – 241.2 μm)	106.0 μm (98.1–117.0 μm).	108.3 μm (97.0 –117.0 μm
*Mentha haplocalyx* Briq. LCW-508	210.9 μm (187.6 – 235.1 μm)	145.1 μm (141.7 – 151.3 μm)	94.1 μm (82.6–106.2 μm)	73.9 μm (65.3 – 88.1 μm)	85.7 μm (69.4–99.1 μm)	29.9 μm (24.3 – 33.9 μm)	184.6 μm (135.4 – 212.1 μm)	135.5 μm (119.7 – 149.5 μm)	83.0 μm (76.0–90.4 μm)	51.3 μm (42.1–63.1 μm)
*Mentha piperata* Briq. LCW-509	151.4 μm (137.6–177.7 μm)	104.5 μm (100.2 – 108.0 μm)	148.4 μm (132.0 – 170.0 μm)	115.2 μm (106.7 – 123.2 μm)	139.2 μm (126.5–153.1 μm)	48.6 μm (41.2–56.3 μm)	142.6 μm (126.3–172.7 μm)	42.0 μm (37.6 – 45.5 μm)	114.6–174.0 μm) 151.8 μm.	158.8 μm 117.0 μm-181.3 μm)
*Tectona grandis* L.f. LCW-510	216.0 μm (198.7 –267.5 μm)	139.9 μm (114.0 – 161.2 μm)	163.0 μm (152.3 – 179.4 μm)	113.9 μm (107.0 – 121.6 μm)	151.8 μm (140.0 -161.7 μm)	45.9 μm (33.6–52.3 μm)	158.0 μm (143.3 – 182.2 μm)	32.4 μm (28.2 – 35.0 μm)	0	0
*Colebrookea opppositifolia* L. LCW-511	146.0 μm (104.5 –171.0 μm)	92.3 μm (59.4–140.2 μm)	128.4 μm (119.9 – 144.7 μm)	76.7 μm (60.2 – 96.9 μm)	127.3 μm (118.9– 139.9 μm)	30.2 μm (26.4 – 36.1 μm)	120.3 μm (111.7–135.1 μm)	49.3 μm (41.7 – 61.0 μm)	0	0
*Galeopsis tetrahit* L. LCW-512	212.1 μm (199.9–233.6 μm)	105.1 μm (82.7–117.7 μm)	107.1 μm (97.5 – 123.3 μm)	85.3 μm (80.2–89.7 μm).	94.2 μm (85.3–104.9 μm)	28.1 μm (23.0– 35.6 μm)	129.7 μm (123.6- 141.3 μm)	41.7 μm (37.1–48.5 μm)	0	0
*Salvia splendens* L. LCW-513	212.4 μm (188.7–241.1 μm)	146.5 μm (141.5–156.0 μm)	150.4 μm (111.0 – 173.3 μm)	109.4 μm (75.2 – 127.1 μm)	116.8 μm (82.0 – 142.0 μm)	38.2 μm (30.1– 47.0 μm)	143.2 μm (141.4 –144.6 μm)	32.3 μm (27.2–37.6 μm)	161.2 μm (155.3–165.1 μm).	156.6 μm (145.2–162.7 μm).
*Salvia officinalis* L. LCW-514	32.7 μm 36.8 μm – 39.7 μm)	149.9 μm (129.6–185.6 μm)	154.6 μm 48.3 μm– 161.9 μm)	131.5 μm (118.3 – 143.4 μm)	155.6µm165.0–162.0 μm)	39.8 μm (35.2 – 46.0 μm)	175.0 μm (130.6–229.2 μm)	50.0 μm (36.1 – 63.0 μm)	0	0
*Salvia rosemarinus* Spenn. LCW-515	280.5 μm (206.0 –360.3 μm)	150.8 μm (77.7–211.9 μm)	166.5 μm (177.4 μm– 167.3 μm`)	128.4 μm (116.2 – 138.2 μm)	155.1 μm (119.9 μm– 194.6 μm	46.9 μm (32.0- 61.3 μm)	189.2 μm (140.8 –234.5 μm)	50.3 μm (43.9 – 59.5 μm)	0	0

**Table 2:** Quantitative anatomical variations among studied Lamiaceae Taxa (Adaxial surface)

Plant Name	Epidermal cell length µm	Epidermal cell width µm	Stomatal cells length µm	Stomatal cells width µm	Guard cells length µm	Guard cell width µm	Subsidiary cells length µm	Subsidiary cells width µm	Trichome length Μm	Trichomewidth µm
*Mentha suaveolens* Ehrh. LCW-501	175.3 μm (148.8–210.2 μm)	116.3 μm (86.8 – 145.0 μm)	0	0	0	0	0	0	69.5 μm (65.2–72.1 μm)	83.0 μm (69.9– 95.6 μm)
*Ajuga reptans* L. LCW- 502	19.8 μm (17.5 – 22.1 μm)	111.1 μm (103.0 – 115.3 μm)	137.2 μm (115.7–151.4 μm)	40.25 μm (55.5 μm – 25 μm).	142.5 μm (130.9 – 151.6 μm)	46.5 μm (43.6 –48.4 μm)	69.85 μm (44.5 – 95.2 μm)	133.2 μm (97.6–188.9 μm)	0	0
*Coleus scutellaroides* L. LCW-503	24.1 μm (20.2–29.8 μm)	98.7 μm (78.6–114.2 μm)	0	0	0	0	0	0	0	0
*Stachys bazantina* K.Koch LCW-504	20.0 μm (19.9 – 20.9 μm)	132.8 μm (125.4 – 143.4 μm)	0	0	0	0	0	0	80.9 μm (77.4–83.9 μm).	78.9 μm (70.7–82.5 μm)
*Origanum vulgare* L. LCW-505	16.1 μm (13.3 – 20.8 μm)	11.9 μm (10.4 – 11.9 μm)	0	0	0	0	0	0	0	0
*Thymus vulgaris* L. LCW-506	155.7 μm (153.4 – 158.2 μm)	95.8 μm (77.1 – 107.9 μm)	0	0	0	0	0	0	0	0
*Oscimum basiculum* L.	19.1 μm (14.4 – 22.8 μm)	85.3 μm (85.1–96.6 μm)	104.6 μm (97.5 – 111.6 μm)	92.9 μm (67.9 – 112.4 μm)	114.4 μm (89.6–156.4 μm)	30.4 μm (26.4 – 35.2 μm)	20.6 μm (12.9–26.3 μm)	21.8 μm (19.4 – 24.2 μm)	34.5 μm (25.7 – 42.7 μm)	57.5 μm (53.0–60.0 μm)
*Mentha haplocalyx* Briq. LCW-508	19.4 μm (18.2 – 20.3 μm)	15.1 μm (11.0 – 18.1 μm).	0	0	0	0	0	0	0	0
*Mentha piperata* Briq. LCW-509	19.7 μm (15.2 – 23.9 μm)	14.3 μm (12.5 – 15.6 μm)	0	0	0	0	0	0	17.4 μm (17.8–18.8 μm)	15.3 μm (14.8–17.3 μm)
*Tectona grandis* L.f. LCW-510	102.9 μm (88.8 μm – 28.3 μm)	150.1 μm (133.5 – 168.6 μm)	0	0	0	0	0	0	0	0
*Colebrookea opppositifolia* L. LCW-511	35.6 μm (28.6 – 40.6 μm)	145.6 μm (130.9 – 159.8 μm)	0	0	0	0	0	0	0	0
*Galeopsis tetrahit* L. LCW-512	243.6 μm (222.0 – 279.3 μm)	140.9 μm (129.4 – 148.0 μm)	135.0 μm (128.2–141.4 μm)	102.0 μm (94.4–111.3 μm)	133.5 μm (122.4- 141.8 μm)	35.3 μm (26.1 μm – 44.8 μm	267.8 μm (221.8 –326.4 μm)	233.8 μm (98.2 – 316.2 μm)	0	0
*Salvia splendens* L. LCW-513	151.9 μm (99.0–192.4 μm)	135.5 μm (87.5–162.7 μm)	0	0	0	0	0	0	148.9 μm (134.3–150.0 μm)	122.9 μm (116.8–127.9 μm).
*Salvia officinalis* L. LCW-514	206.8 μm (169.9–225.5 μm)	144.6 μm (127.3 – 157.6 μm)	0	0	0	0	0	0	55.2 μm (36.6 – 94.7 μm)	78.8 μm (60.5 – 98.2 μm)
*Salvia rosemarinus* Spenn. LCW-515	30.2 μm (27.5–32.6 μm)	15.3 μm (12.8 – 17.7 μm).	129.9 μm (118.6 – 150.6 μm)	119.1 μm (92.4–140.0 μm)	140.4 μm (119.0 – 157.5 μm)	34.7 μm (28.4 – 39.8 μm).	20.4 μm (19.3 – 23.5 μm)	64.4 μm (54.7 – 77.3 μm).	93.5 μm (70.2 μm – 15.0 μm)	108.9 μm (102.5–117.3 μm)

### Shape of epidermal cells

**Fig. 1:**
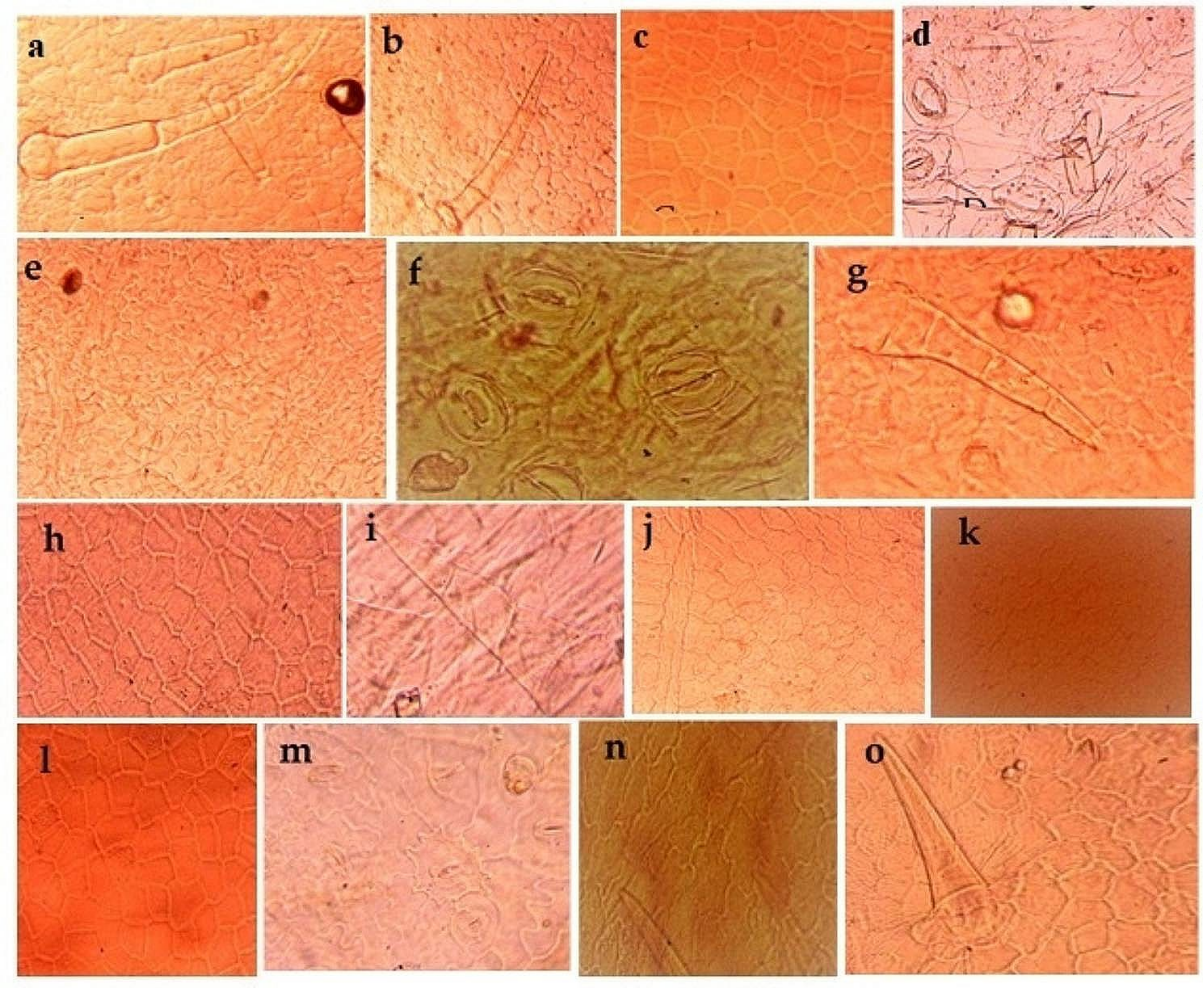
**a;** pentagonal straight-walled epidermal cells and diacytic stomata in *Mentha suaveolens*. **b;** irregular sinuous shaped epidermal cells and diacytic stomata in *Ajuga reptans*. **c;** hexagonal epidermal cells in *Coleus scutellaroides*. **d;** pentagonal three-dimensional epidermal cells and long tubular trichomes in *Stachys byzantina*. **e;** oblong sinuous polygonal epidermal cells and actinocytic stomata in *Origanum vulgare*. **f;** polygonal epidermal cells in *Thymus vulgaris*. **g;** square, polygonal cell, peltate trichomes, and paracytic stomata in *Oscimum basilicum*. **h;** irregular polygonal shape epidermal cells and anisocytic stomata in *Mentha haplocalyx*. **i;** irregular, folded, or plicate walled epidermal cells and actinocytic stomata in *Mentha piperata*. **j;** irregular, pentagonal epidermal cells with thin and sinuous anticlinal walls in *Tectona grandis*. **k;** sinuous shape epidermal cells and anisocytic or actinocytic stomata in *Colebrookia oppositifolia*. **l;** irregular polygonal shape epidermal cells and anisocytic or paracytic stomata in *G. tetrahit*. **m;** polygonal and hexagonal epidermal cells in *Salvia splendns*. **n;** sinuous shape epidermal cells in *Salvia officinalis*. **o;** Abaxial view LM showing sinuous shape epidermal cells and paracytic stomata *Salvia rosemarinus*

The diversity in the shapes of epidermal cells were observed. Typically, species exhibit hexagonal and pentagonal epidermal cells characterized by straight anticlinal wall thickness. Whereas, some species have irregular epidermal cells with sinuous or wavy anticlinal wall thickness. On abaxial surface, pentagonal and straight walled epidermal cells were mainly found in *Mentha suaveolens*. Irregular shaped cells were seen in *Ajuga reptans*, *Colebrookea oppositifolia, Galeopsis tetrahit, Salvia offcinalis, Salvia rosemarinus and Tectona grandis* while polygonal cells were observed in *Coleus scutellaroides, Origanum vulgare, thymus vulgaris, oscimum basiicum*, however cells of *Mentha haplocalyx, salvia splendens* and *Mentha piperata* were reported as hexagonal. On adaxial surface, polygonal cell shape was most commonly observed in *C. scutellaroides*, *M. haplocalyx, M. piperata, Tectona grandis* and *G. tetrahit*. Hexagonal cells were observed in *S. bazyntina*, some species such as *O. vulgare* and *S. splendens* showed mosaic shaped epidermal cells (Fig. 1).

### Anticlinal wall thickness

Most of the species showed straight walled anticlinal thickness whereas, some species such as *S officinalis, S rosemarinus, C oppositifolia, T grandis*, and *Ajuga reptans* showed sinuous or folded anticlinal wall thickness. In adaxial view of epidermis, species such as *A. reptans, O. vulgare, C. oppositifolia, S. splendens, S. officinalis, S. rosemarinus* showed sinuous or folded anticlinal wall thickness whereas, other species showed straight walls.

### Epidermal cells size

In the adaxial view, the length of epidermal cells ranged from 280.5 ± 1.25 to 10.9 ± 0.45 μm. *S. rosmarinus* exhibited the largest size, while O. vulgare had the smallest. The average width of epidermal cells varied from 21.9 ± 1.75 to 82.7 ± 1.35 μm, with *M. suaveolens* displaying the widest and *T. vulgaris* the narrowest. In the abaxial view, the cell length ranged from 3247 ± 14 to 10.8 ± 0.85 μm. *S. officinalis* possessed the largest cell size, while *O. vulgare* showed the smallest. Regarding width, in the abaxial view, *M. haplocalyx* exhibited the largest average width of 15.1 μm, while *O. basilicum* had the smallest width of 85.3 μm. Furthermore, the largest average length ranged from 35.6 μm to 15.9 μm, with *C. oppositifolia* displaying the largest and *S. splendens* the smallest average length.

### Types of stomata cells

In this study, various stomatal types were observed across different species of Lamiaceae leaves. These types include paracytic, anisocytic, diacytic, and anomocytic. On the abaxial surface, paracytic stomata were predominantly found in *S. officinalis, S. splendens, S. rosmarinus, G. tetrahit, T. grandis, M. haplocalyx*, and *T. vulgaris*. However, anisocytic stomata were noted in *C. scutellaroides, O. basilicum, M. piperata, and C. oppositifolia*. Diacytic stomata were observed in *M. suaveolens* and *A. reptans*. Additionally, *S. officinalis* and *S. rosmarinus* exhibited some anomocytic stomata. On the adaxial surface, paracytic stomata were found in *O. basilicum* and *S. rosmarinus*, while diacytic stomata were observed in *A. reptans*. Anisocytic stomata were noted in *G. tetrahit*.

### Size of guard cells

**Fig. 2:**
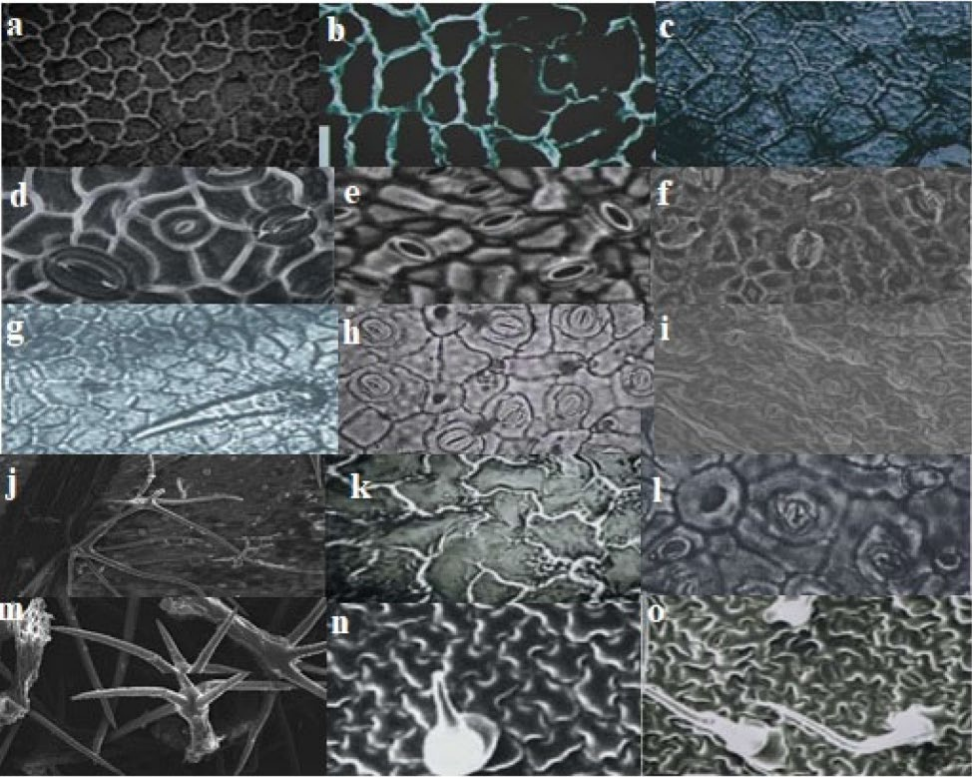
**a;** sinuous shaped epidermal cells of *M. suaveolens*. **b;** sinuous epidermal cells of *A. repentance*. **c;** hexagonal epidermal cells of *C. scutellaroides*. **d;** anomocytic stomata and hexagonal epidermal cells in *S. byzantina*. **e;** actinocytic stomata in *O. vulgare*. **f;** paracytic stomata and polygonal shaped epidermial cells in *T. vulgaris*. **g;** trichomes with acute apex and polygonal epidermal cells in *O. basilicum*. **h;** polygonal epidrmal cells and paracytic stomata in *M. haplocalyx*. **i;** pilcate walled epidermal cells and actinocytic stomata in *M. piperata*. **j;** irregular sinuous anticlinal walls in *T. grandis*. **k;** sinuous epidermis and anisocytic stomata in *C. oppositifolia*. **l;** anisocytic stomata and polygonal epidermal cells in *G. tetrahit*. **m;** short non-glandular tapering trichomes in *S. splendens*. **n;** long tubular trichomes and sinuous epidermis in *S. officinalis*. **o;** Non-glandular trichomes with acute apex and sinuous anticlinal wall thickness in *S. rosemrinus*

Various sizes of guard cells observed in the Lamiaceae family are outlined. In the abaxial view, the average length of guard cells ranged from 85.7 ± 0.45 to 155.6 ± 1.25 μm. *S. officinalis* displayed the longest guard cells, whereas *M. haplocalyx* exhibited the shortest. The average width of guard cells ranged from 28.1 ± 0.25 to 64.5 ± 1.45 μm, with *M. suaveolens* possessing the widest guard cells and *G. tetrahit* having the narrowest. In the adaxial view, guard cell length varied from 114.4 ± 1.69 to 142.5 ± 1.07 μm. *A. reptans* had the largest average length of guard cells, while *O. basilicum* displayed the smallest. The average width of guard cells ranged from 30.4 ± 1.13 to 46.5 ± 1.69 μm, with A. *reptans* showing the widest guard cells and *O. basilicum* exhibiting the narrowest (Fig. 2).

### Size of subsidiary cells

In abaxial view, average length of subsidiary cells ranged from 104.9 ± 0.45 to 295.7 ± 0.75 μm. *A reptans* showed largest length of subsidiary cells and *O. vulgare* had smallest length of subsidiary cells. Average width of subsidiary cells ranged from 30.9 ± 1.25 to 216.8 ± 1.75 μm. *O. basiicum* had largest width of subsidiary cells and *O. vulgare* showed smallest width of subsidiary cells. In adaxial view, average length of subsidiary cells ranged from 267.8 ± 1.47 to 161.2 ± 1.15 μm. *G tetrahit* had largest length of subsidiary cells and *A. reptans* had smallest length of subsidiary cells. Average width of subsidiary cells ranged from 133.2 ± 1.95 to 233.8 ± 1.95 μm. *G. tetrahit* had largest width of subsidiary cells and *A. reptans* had smallest width of subsidiary cells.

### Trichome types

In the present study, trichomes were present in many species. On abaxial surface trichomes were present in *M. suaveolens, A. reptans, T. vulgaris, M. haplocalyx, S. splendens*, in rest of species trichomes were absent on abaxial surface. In adaxial side trichomes were present in *M. suaveolens, A. reptans, S. bazyntina, O. basiicum, S. splendens, S. officinalis, S. rosemarinus*. These trichomes were long, non-glandular, unbranched and having acute apex. In rest of selected species trichomes were absent on the adaxial side. *Salvia officinalis* species showed a higher trichome density on the adaxial surface than *Mentha piperata.* These anatomical chacracters can be used as diagnostic tools for identifying species within genera.

### Size of trichomes

**Fig. 3:**
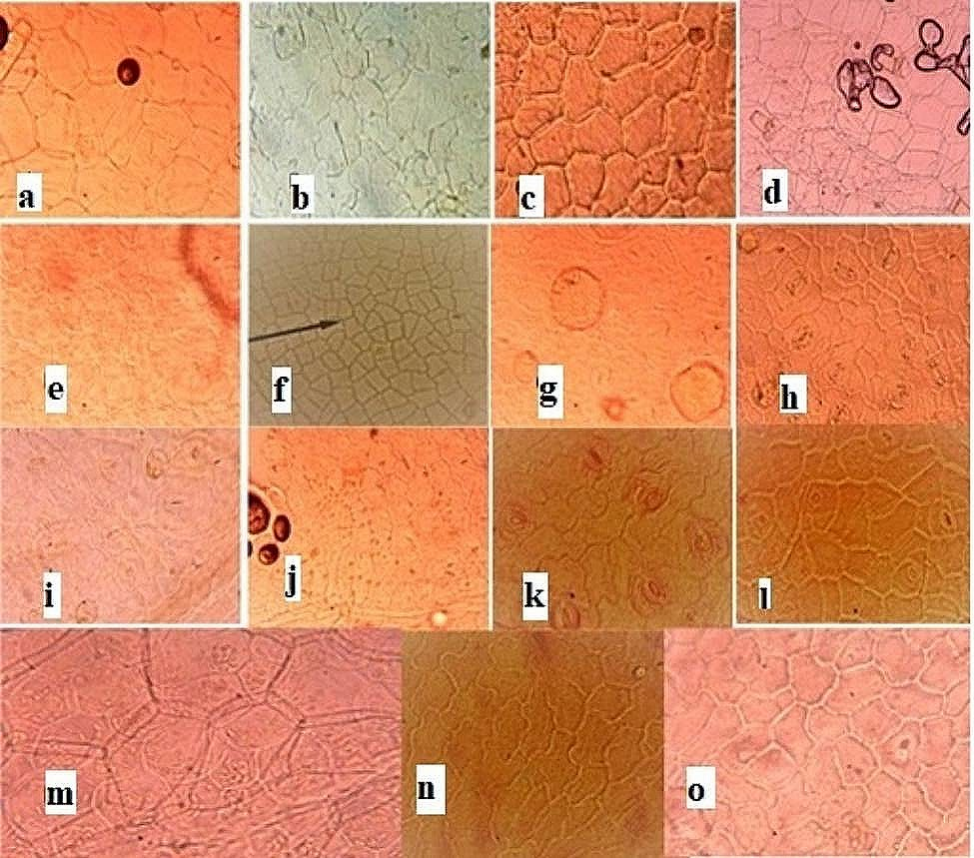
**a;** long, tubular, tapering trichomes in *Mentha suaveolens*. **b;** sinuous shape epidermal cells, long tubular trichomes in *Ajuga reptans*. **c;** polygonal, straight walled epidermal cells in *Coleus scutellaroides*. **d;** Ano-mocytic stomata in *Stachys byzantina*. **e;** polygonal oblong, jigsaw-shaped epidermal cells in *Origanum vulgare*. **f;** anisocytic or paracytic stomata in *Thymus vulgaris*. **g;** oil droplets, trichomes with the acute apex in *Oscimum basilicum*. **h;** oblong or square-shaped hexagonal epidermal cells in *Mentha haplocalyx*. **i;** short-length tubular having tapering end trichome is shown in *Mentha piperata*. **j;** polygonal shape epidermal cells in *Tectona grandis*. **k;** sinuous shape epidermal cells in *Colebrookia oppositifolia*. **l;** polygonal and irregular shape epidermal cells in *G tetrahit*. **m;** showing polygonal and irregular epidermal cells having plicate walls, paracytic and diacytic stomata *Salvia splendns*. **n;** sinuous shape epidermal cells and long tubular trichomes with acute apex in *Salvia officinalis*. **o;** sinuous shape epidermal cells and long, non-glandular trichome with acute apex *Salvia rosemarinus*

In abaxial view, average length of trichomes ranged from 67.9 ± 1.25 μm to106.0 ± 1.25 μm. *M. suaveolens* had largest length of trichome and *O. basiicum* had smallest length of trichome. Average width of trichome ranged from 48.2 ± 1.75 to 16.6 ± 1.45 μm. *S splendens* had largest width of trichome while *T. vulgaris* had smallest trichome width. In adaxial view *S. rosemarinus had largest average length* and *S. bazyntina* had smallest average length. Lengths observed were 89.5 ± 1.65 and 80.9 ± 1.35 μm, respectively. Average width observed was 44.5 ± 1.35 and 158.3 ± 1.45 μm, *A. reptans*. has smallest and *M. piperata* had the largest average width of trichomes (Fig. 3).

### Correlation analysis

**Fig. 4:**
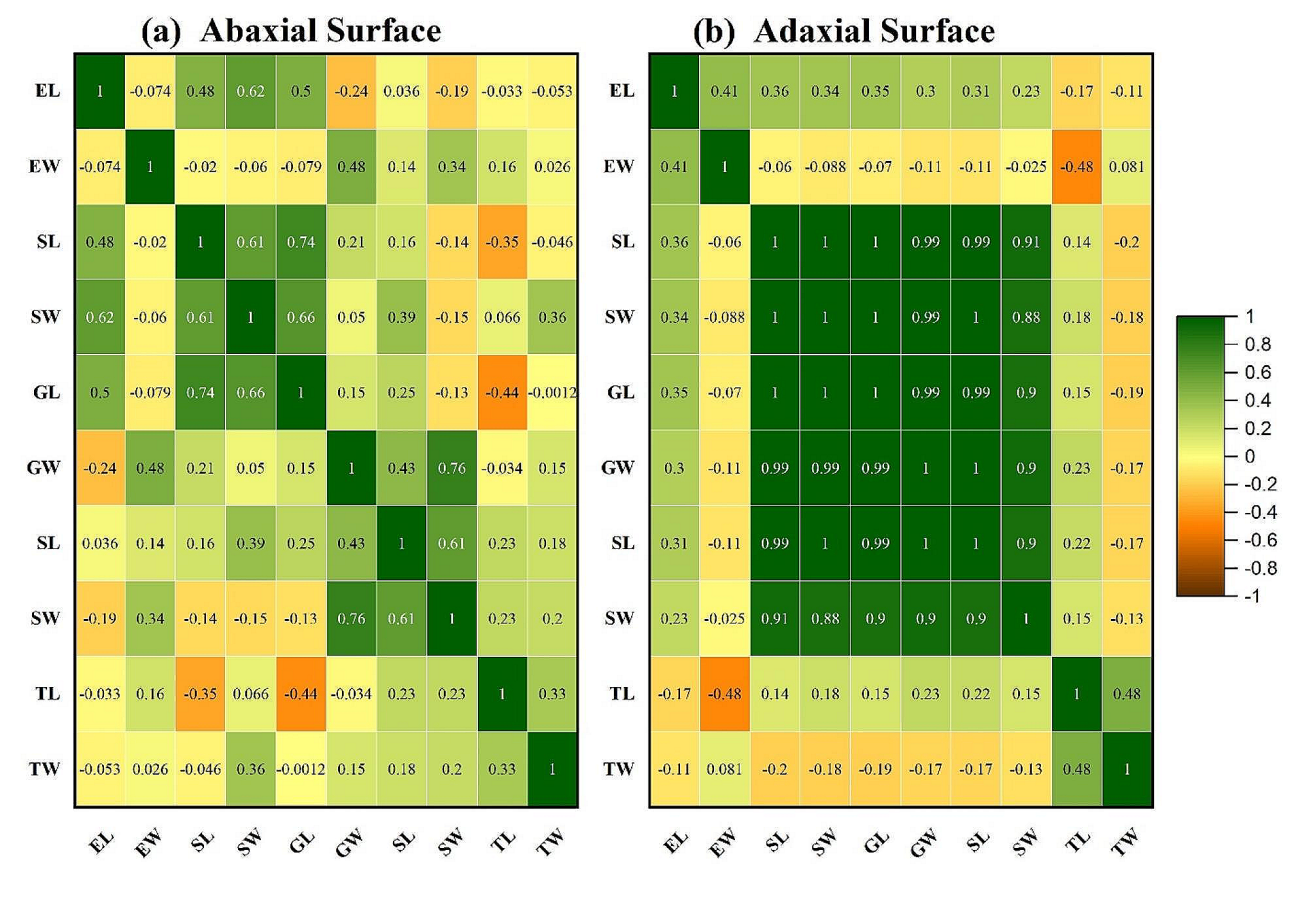
Principal component analysis Biplot of abaxial and adaxial surface of leaf. LT: Trichome length, WT: Trichome width, WE: epidermal cell width, WSB: width of subsidiary cell, WG: Guard cell width, LSB: length of subsidiary cells, WS stomatal width, LG: length of guard cells, LE: length of epidermal cells

**Fig. 5:**
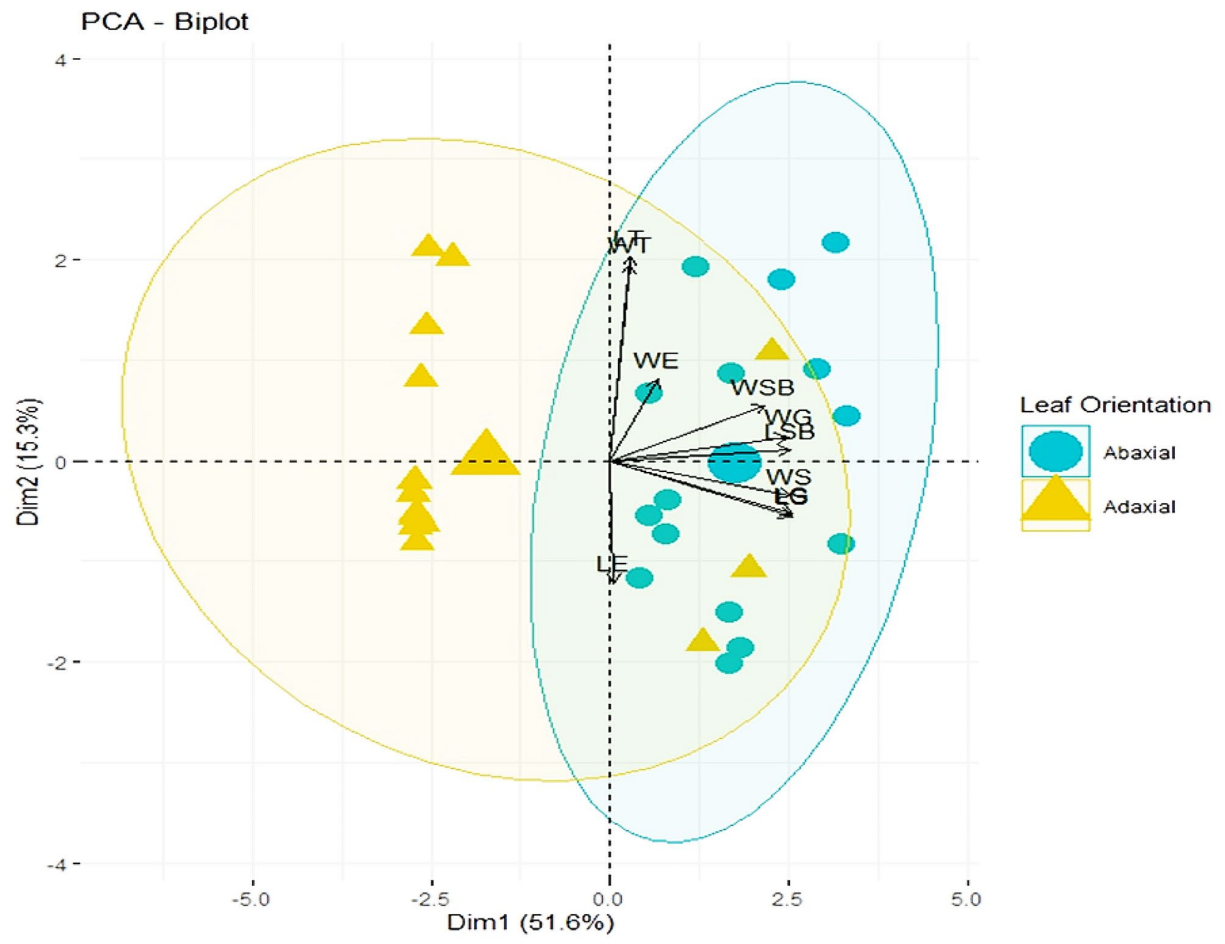
Correlation chart showing the significance of association between anatomical characteristics of the abaxial (**a**) and adaxial surface (**b**) of studied samples. EL: epidermal cell length, EW: epidermal cell width, SL: stomata length, SW: stomatal width, GL: guard cell length, GW: Guard cell width, SL.1: subsidiary cell length, SW.1: subsidiary cell width, TL: trichome length, TW; Trichome width

Correlation on the abaxial side in SL, SW has a significant positive relationship in the same direction as GL, and GW. TL has a positive correlation with TW in the same direction. SL. 1 has a negative correlation with SW.1. SW, GL, GW have a strong positive significant correlation with SL.1 and SW.1 in the same direction (Fig. 4). On the adaxial side, SW, GL has a positive correlation in the same direction with the SW1.EL.GW and GL have a significant correlation with the SL1 and TL in the same direction (FIgur 4). On adaxial side, SW, GL has positive correlation in the same direction with the SW1.EL.GW, GL has significant correlation with the SL1 and TL in the same direction (Fig. 5).

### Principle component analysis

The above figure shows the mean values of length and width of epidermal cells, stomata, trichomes, guard cells, and subsidiary cells on the abaxial and adaxial surface of leaves in collected samples of the family Lamiaceae. The highest correlation is observed for trichomes length only (Fig. 4). Principal component analysis revealed significant differences between the different parameters. PC1 and PC2 accounted for 66.9% of the variation in the distribution of data within the biplot.

## Discussion

In the present research work the anatomical features observed in studied Lamiaceae taxa agreed with those discussed by Tarimcilar et al. [9]. The present research work focuses the important leaf epidermal anatomical features which are helpful in differentiation and identification of plant species. This study presents important information about the microscopic structure of the leaf surface, specifically the various types of trichomes on the leaves of plants in the Lamiaceae family. Additionally, this study contrasts these observations with microscopic examinations. A wide range of trichomes, encompassing both glandular and non-glandular types, were discovered on both the vegetative and reproductive parts of Lamiaceae plants. This research offers a remarkably thorough examination of trichome micromorphology within the Lamiaceae family. The various types of trichomes distinguished in this study notably augment the significance of these characteristics in the family’s taxonomy. The results affirm the utility of these traits in delineating challenging taxa and facilitate the demonstration of systematic relationships among the species. Previous studies of trichomes morphology in Lamiaceae have also highlighted the significance of these traits [10–15].

Foliar epidermal variation indicating environmental plasticity was observed [16, 17]. Cells of *Stachys byzantine* showed three dimensional, pentagonal and cuboidal symmetry with straight double walled epidermal cells by Pachkore et al. [17] stated similar resuts for *Mentha piperata* and *Mentha spicata.* As noted by Hallahan [18], the shapes and sizes of epidermal cells on both the upper and lower surfaces of leaves play a crucial role in taxonomy. Within the Lamiaceae family, leaf epidermal characteristics exhibit notable diversity in terms of epidermal cell shapes, stomatal complexes, and patterns of anticlinal walls, which may be influenced by ecological factors.

Metcalfe and Chalk [19] reported that within the Lamiaceae family, the diacytic type of stomata is the most commonly found, often mixed with the anomocytic type. El- Gazzar and Watson [20] also conducted the diverse of stomatal arrangements present in Lamiaceae, our investigation unveiled the prevalence of the anisocytic type, anomocytic, diacytic, anisocytic, or a blend of anomocytic and anisocytic stomata. Through our observations, we have determined that leaves of Lamiaceae predominantly feature diacytic, normocytic, and anisocytic stomata. Additionally, anisocytic and tetracytics stomata have also been found. Abdulrahaman and Oladele [21] also reported anisocytic diacytic and paracytic stomata in some *Origanum* species. All the trichomes were long, unbranched non glandular, having tapering ends. The number, distribution, and micromorphology of glandular trichomes can serve as distinguishing features at the subfamily level of Lamiaceae [22].

Whereas, in *S. splendens* peltate and glandular trichomes were also seen Kahraman et al. [23] also reported glandular and peltate trichomes in *Saliva chrysophylla*. NGTs were classified as simple unicellular and multicellular based on cell number and morphology. *T. grandis* had non glandular, simple and branches trichomes. Haruna and Ashir [24] also showed similar results for *T. grandis*. identified abundant non-glandular trichomes in *L. dentata*, alongside some peltate and capitate Type 1 glandular trichomes. Within the Lamiaceae family, non-glandular trichomes are more commonly found than glandular trichomes and can be categorized based on their morphology, dimensions, cell count, wall thickness, and ornamentation. These characteristics have served as valuable taxonomic markers for plant taxonomists in species identification and differentiation.

The study conducted by Gul et al. [25] was very helpful in identifying and delimitating the Lamiaceae species at the tribe and species level, through the use of SEM. SEM involves high magnification, which enables the detection of micromorphological variation that is not visible under LM in other groups of plants, as noted by Xiang et al. [26] and Yuan et al. [27].

## Conclusion

Recent studies are taxonomically valuable having significant morpho-anatomical features. Both qualitative and quantitative measurements were conducted including trichomes, stomata, epidermal cells and subsidiary cells present on both the abaxial and adaxial epidermis. Significant anatomical differences were noted among these species. Most species had pentagonal and hexagonal epidermal cells having straight anticlinal wall thickness. In *S. splendens* peltate and glandular trichomes were seen whereas in other species trichomes long and unbranched non- glandular, having tapering ends. In adaxial side, trichomes were present only in *M. suaveolens, A. reptans, S. bazyntina, O. basciculum, S. splendens, S. officinalis* and *S. rosemarinus*. These findings affirm the taxonomic usefulness of anatomical traits in identifying the examined Lamiaceae taxa. Also this study is a revision and upgradation of flora of pakistan as no detailed information of these Lamiaceae taxa already exists in flora of Pakistan.

## Materials and methods

### Collection of selected specimens

**Fig. 6:**
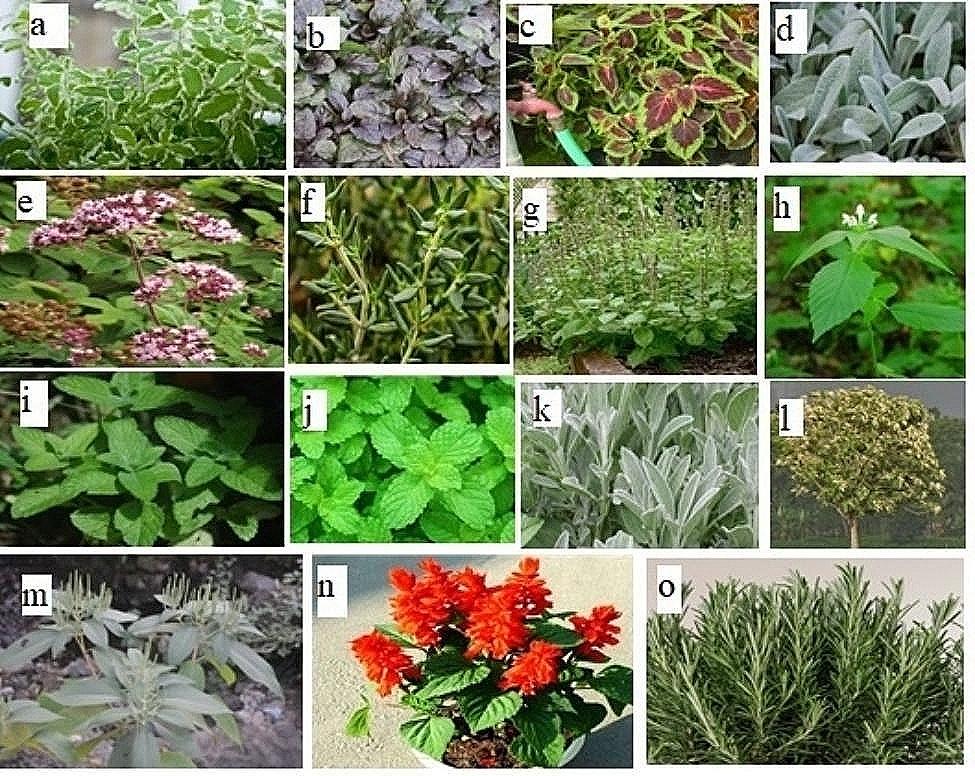
(**A**) *Mentha suaveolens* (**B**) *Ajuga reptans* (**C**) *Coleus scutellaroides*. (**D**) *Stachys byzantina.* (**E**) *Origanum vulgare*. (**F**) *Thymus vulgaris*. **G***Oscimum basilicum*. **H**. *Galeopsis tetrahit*. **I**. *Mentha haplocalyx*. **J**. *Mentha piperata*. **K**. *Tectona grandis*. **L**. *colebrookia oppositifolia*. **M**. *Salvia splendens*. **N**. *Salvia officinalis***O**. *Salvia rosemarinus*

Fresh fifteen specimens of family lamiaceae were collected by visiting different nurseries in Lahore. Collected samples were subjected to identification by consulting different herbaria and floras. [6, 7]. Further studies were carried out in the Plant Taxonomy Laboratory of the Botany department of Lahore College for Women University (Fig. 6).

### Preservation and herbarium preparation of samples

Collected plant material was kept and pressed in layers of newspaper for drying. Newspaper was changed after intervals to avoid fungal attack. When dried thoroughly, plants were transferred to herbarium sheets and labelled. Fresh leaves were used for light and scanning electron microscopy.

### Anatomical study

The epidermal leaf anatomy of the plant species was examined to observe the diversity among different species, utilizing both light and scanning electron microscopy. The leaves underwent a process involving boiling in a solution of 70% hot lactic acid and 30% ammonia for 50–60 min until they were suitable for epidermal scraping. Slides were then prepared according to the methods outlined by Cotton (1974) and Clark (1960) with slight modifications. For the preparation of the abaxial surface, the leaf was positioned on a tile with its adaxial surface facing upwards and flooded with 70% cold lactic acid. Subsequently, it was carefully peeled to ensure that the transparent membrane of the leaf remained on the tile. Immerse the leaf samples in the staining solution safranine for the recommended duration, typically a few minutes to several hours. After staining, rinse the leaf samples in distilled water to remove excess stain and prevent background staining. Transfer the stained leaf samples onto glass microscope slides using fine forceps. Apply a mounting medium such as glycerol to preserve the samples and prevent dehydration. Place a coverslip over the mounted leaf sample using a mounting medium to secure it in place. Remove any air bubbles trapped under the coverslip by gently pressing down on the edges. Allow the mounting medium to dry completely before sealing the edges of the coverslip with nail polish or a commercial sealant to prevent evaporation and specimen damage. Examined the stained leaf samples under a light microscope (Figs. 1, 2 and 3).

### Scanning electron microscopy

Select healthy leaves from the plant species. Immerse the collected leaves in a fixative solution immediately after harvest to preserve their structure. Common fixatives include glutaraldehyde or formaldehyde in a buffered solution. Mount the dried leaves onto SEM stubs or holders using conductive adhesive or double-sided carbon tape. Ensure secure attachment to prevent sample movement during imaging. Transfer the mounted sample stubs into the SEM chamber and ensure proper alignment. Adjust SEM parameters such as accelerating voltage, beam current, working distance, and aperture size based on the sample type and imaging requirements.

### Microscopic analysis

Light and scanning electron microscope was used to critically analyze the leaf epidermis of each species. The epidermal features included epidermal cells, the shape of guard cells, the number of subsidiary cells, types of trichomes and stomatal shape were analyzed (Fig. 5).

### Statistical analysis

To investigate the statistical relationships among diverse morpho-anatomical parameters, the data were organized in an Excel spreadsheet format. Principal Component Analysis (PCA) was employed to illustrate the interrelationships among these parameters and the leaf surface [28]. Subsequently, the Pearson correlation coefficient was computed to quantify the associations between different parameters. The resultant correlations were visually depicted in a correlogram using the ‘corrplot’ package [29].

## Data Availability

Data is contained within the article.
